# Synergistic Effects of Boron and Rare Earth Elements on the Microstructure and Stress Rupture Properties in a Ni-Based Superalloy

**DOI:** 10.3390/ma17092007

**Published:** 2024-04-25

**Authors:** Qiang Tian, Shuo Huang, Heyong Qin, Ran Duan, Chong Wang, Xintong Lian

**Affiliations:** 1 Central Iron and Steel Research Institute Co., Ltd., Beijing 100081, China; qtian1frank@163.com (Q.T.); qinheyong@126.com (H.Q.); duanran@cisri-gaona.com.cn (R.D.); 2 School of Materials Science and Engineering, Northeastern University, Shenyang 110819, China; 2190025@stu.neu.edu.cn; 3 School of Materials Science and Engineering, Shanghai University, Shanghai 200444, China

**Keywords:** Ni-based superalloy, boron, rare earth, element segregation, stress rupture life

## Abstract

The synergistic effects of boron (B) and rare earth (RE) elements on the microstructure and stress rupture properties were investigated in a Ni-based superalloy. The stress rupture lifetime at 650 °C/873 MPa significantly increased with the addition of B as a single element. Furthermore, the stress rupture lifetime reached its peak (303 h), with a certain amount of B and RE added together in test alloys. Although the grain size and morphology of the γ′ phase varied a little with the change in B and RE addition, they were not considered to be the main reasons for stress rupture performance. The enhancement in stress rupture lifetime was mostly attributed to the segregation of the B and RE elements, which increased the binding force of the grain boundary and improved its strength and plasticity. In addition, the enrichment of B and RE inhabited the precipitation of carbides along grain boundaries. Furthermore, nano-scale RE precipitates containing sulfur (S) and phosphorus (P) were observed to be distributed along the grain boundaries. The purification of grain boundaries by B and RE elements was favorable to further improve the stress rupture properties.

## 1. Introduction

Nickel-based superalloys have been considered as a kind of promising metal for high-temperature structure components in the field of aerospace, especially used for gas turbine components due to their outstanding comprehensive mechanical properties [1,2,3,4]. Superalloys, such as GH4169, GH4706 and GH4742, are commonly used in gas turbine engines under the conditions of extreme heat exposure. Among these superalloys, the GH4742 alloy is a typical Ni-Cr-Co-based superalloy, strengthened by the precipitation of the γ′ phase with a high content of Al, Ti and Nb. The total content of alloying strengthening elements is more than 7.8%, which are the forming elements of the γ′ phase. After heat treatment, the content of the γ′ phase can reach 35%. Moreover, the GH4742 alloy has good high-temperature properties due to the solution-strengthening effect of Mo, Co and the grain-strengthening effect of microalloying elements like rare earth elements and B. Based on its special chemical components, the strength of the GH4742 alloy can exceed 1300 MPa under room temperature and 1000 MPa under 700 °C. The process of the GH4742 alloy usually consists of double vacuum melting (VIM+VAR), forging cogging and hot die forging forming, which can ensure the purity of this material. Therefore, the GH4742 alloy has been widely used in high-temperature components, for instance, high-pressure turbine discs under a service temperature of 750–800 °C [5,6].

As technology develops, new-type gas turbines put forward higher service performance requirements for their rotating high-pressure turbine discs. The service life of the GH4742 alloy high-pressure turbine disc is expected to be increased from more than 50 h at 650 °C/823 MPa to more than 50 h at 650 °C/873 MPa without increasing the upper limit of hardness. It is known that three traditional methods can be used to improve the service life, including controlling the grain size by reasonably forging and the use of heat treatment, strengthening the precipitated phase with a certain size and distribution through the heat treatment system and strengthening the grain boundary by microalloying [7,8,9,10]. Among these, recent studies have concentrated on optimizing superalloys by employing the potential precipitation and solute effect of microalloying elements, which makes it possible to precisely control the microstructure and maximally optimize the performance. For instance, B is one of the most widely used trace elements in superalloys, with remarkable influence on prolonging long-time properties, such as stress rupture and creep life, and inhibiting fatigue crack propagation [11,12,13]. Researchers have found that the segregation of boron (B) can decrease the energy of the grain boundary and affect its diffusion [14,15]. Previous studies have also investigated the addition of B in some Ni-based superalloys, which can modify the morphologies and distribution precipitates along grain boundaries, such as the δ phase, η phase, carbides or even Laves phase, thus enhancing the stress rupture properties [16]. Recently, rare earth (RE) elements have been regarded as promising alloying elements for nickel-based superalloys due to their effects on strengthening the grain boundary and promoting solid solution strengthening [17,18,19]. RE elements have been applied successfully in many metallurgy fields to improve the mechanical performance of materials and oxidation resistance properties of nickel-based superalloys [20,21,22,23].

In this study, the synergistic effects of B and RE elements on the microstructure and stress rupture properties were investigated and discussed. The existing forms of these trace elements in the alloy were further studied and confirmed accurately by the utilized characterization methods of transmission electron microscope (TEM), electron probe microanalysis (EPMA) and Auger electron spectroscopy (AES). This study concludes with a brief discussion of the experimental results, which will establish the theoretical basis of B and RE microalloying and provide guidance for the application of GH4742 alloy or other types of superalloys.

## 2. Materials and Methods

The chemical compositions (weight, %) of GH4742 alloy used in this study were detected by SPECTRO MAX 6 (Kleve, Germany) direct-reading spectrometry, as shown in Table 1. The ingots with a diameter of 150 mm were obtained by vacuum induction melting (VIM) and vacuum arc remelting (VAR). After homogenizing for 72 h at 1135 °C and forging at 1100 °C, a certain size of turbine disc forging was prepared. Then, being treated through heat treatment processes of 1120 °C × 8 h (air cooling) + 780 °C × 16 h (air cooling), the samples were evaluated by means of microstructure characterization and mechanical property testing.

The microstructure was measured by Olympus GX71 (Tokyo, Japan) optical microscope (OM), JEOL JSM-7800F (Tokyo, Japan) scanning electron microscope (SEM) and FEI Talos F200X (Waltham, MA, USA) transmission electron microscope (TEM) to observe the evolution of grain and precipitation. Image-Pro Plus 6.0 software was used for quantitative statistics of grain and second phase, and the number of γ′ phases was more than 100. The samples were etched using a solution of 20 g CuCl_2_ + 100 mL HCl + 100 mL C_2_H_5_OH for OM observation. The SEM sample was electrolytically polished at 20 V for 10–20 s in a solution of 80 mL CH_3_OH + 20 mL H_2_SO_4_, followed by a solution of 15 g Cr_2_O_3_ + 10 mL H_2_SO_4_ + 150 mL H_3_PO_4_ for electrolytic etching. For TEM observation, the sample was ground down to a thickness of less than 50 μm by sandpaper and electrolytically thinned with 70 mA in a solution of 10% HClO_4_ alcohol at a temperature of −30 °C.

JEOL JXA-8230 (Tokyo, Japan) Electron probe microanalysis (EPMA) was conducted to quantitatively analyze the composition gradient near grain boundaries. The ULVAC PHI710 (Chigasaki, Japan) in situ scanning Auger electron spectroscopy (AES) was adopted to measure the change in element concentration. The sputtered electron beam voltage was 10 kV and the current was 10 nA. The ion beam (energy of 2 keV) scanned an area of 2 mm × 2 mm, and the interval was half a minute.

**Figure 1:**
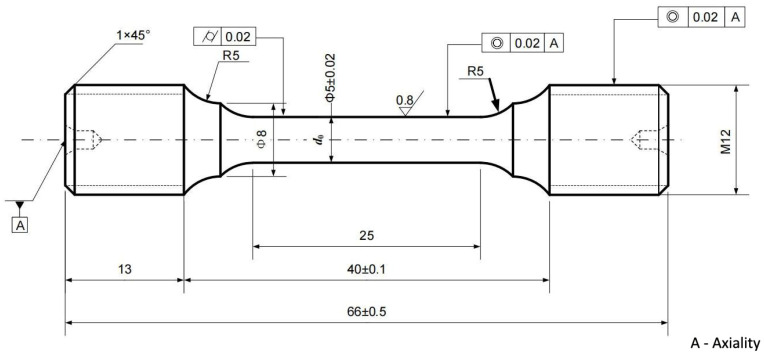
Schematic diagram for stress rupture specimens (mm).

The stress rupture specimens were fabricated from the rim of discs prepared with 60 mm thickness, 200 mm gauge width and 200 mm gauge length along chordwise direction, as shown in Figure 1. The stress rupture tests were conducted on a computer-controlled machine at temperature of 650 °C under stress of 873 MPa according to the standard of GB/T 2039-2012 [24]. The characteristics of fracture surface morphology were examined by SEM.

## 3. Results

### 3.1. Effects of B and RE Elements on Microstructure

**Figure 2:**
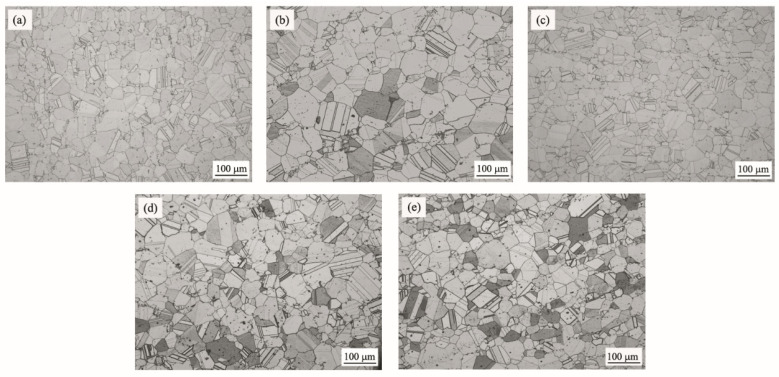
Microstructure of different GH4742 test alloys: (**a**) alloy 1; (**b**) alloy 2; (**c**) alloy 3; (**d**) alloy 4; (**e**) alloy 5.

Figure 2 shows OM images of different GH4742 test alloys. The grains were all determined as a homogeneous austenitic matrix with a large number of twins. Calculated by Image-Pro Plus software, the average grain size of alloy 1 to alloy 5 was determined as 70.7 um, 101.8 um, 67.8 um, 87.3 um and 78.7 um, respectively. There was no regularity between the variation in average grain size and the contents of B and RE. Due to the high alloying of the GH4742 alloy, a complex deformation process is required, which results in unstable control of thermal deformation and causes a dramatic change in grain size according to a previous study [15,23]. There, the change in the microstructure on test alloys probably had little relationship with the addition of contents of B and RE.

**Figure 3:**
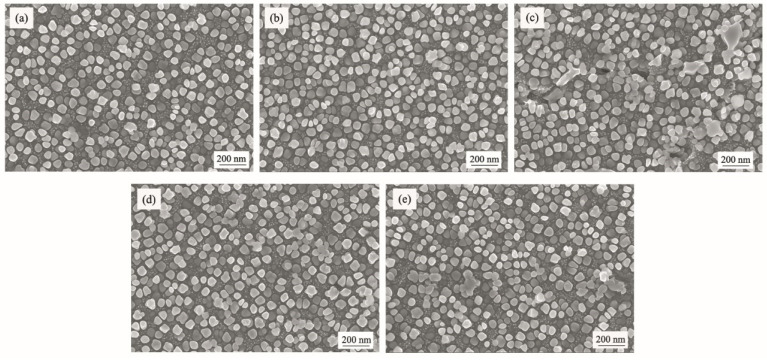
SEM images of γ′ phase of different GH4742 test alloys: (**a**) alloy 1; (**b**) alloy 2; (**c**) alloy 3; (**d**) alloy 4; (**e**) alloy 5.

The SEM analysis indicated that the microstructure consisted of γ matrix and γ′ precipitates, as shown in Figure 3. It can be seen that large amounts of the square primary γ′ phase were observed to precipitate inner grains, while the irregular primary γ′ phase tended to be precipitated along grain boundaries in all test alloys. Small-sized spherical γ′ particles were found to be located between square primary γ′ phases, which were likely to be precipitated during the subsequent aging treatment. From the SEM results above, the addition of B and RE seemed to have no significant effects on the size and morphology of the γ′ phase.

**Figure 4:**
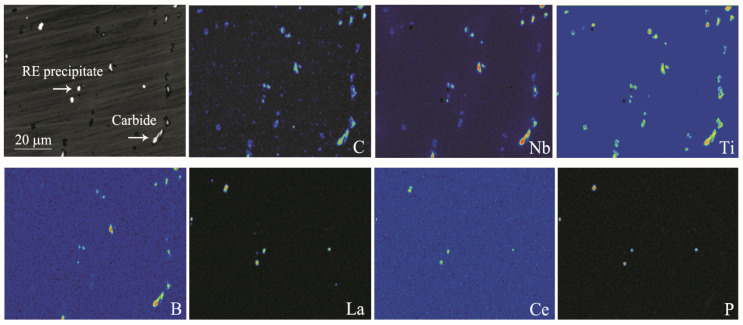
EPMA images of element distribution in precipitate towards grain boundary in alloy 5.

In order to show the synergistic effects of RE and B on precipitates, EPMA was applied to observe the morphology and measure the element distribution of precipitates. The results in test alloy 5 are shown in Figure 4. Large amounts of carbides containing C, Nb, Ti and B elements were observed to be precipitated at the grain boundaries. In addition, fine spherical or ellipsoidal RE compounds containing La, Ce and P elements were found to be distributed with a size of less than 2 μm near the grain boundaries. The results above showed that after B and RE alloying, B and RE may combine with small amounts of other elements to form more complicated oxides, sulfides or complex inclusions.

### 3.2. Effects of B and RE Elements on Stress Rupture Properties

**Figure 5:**
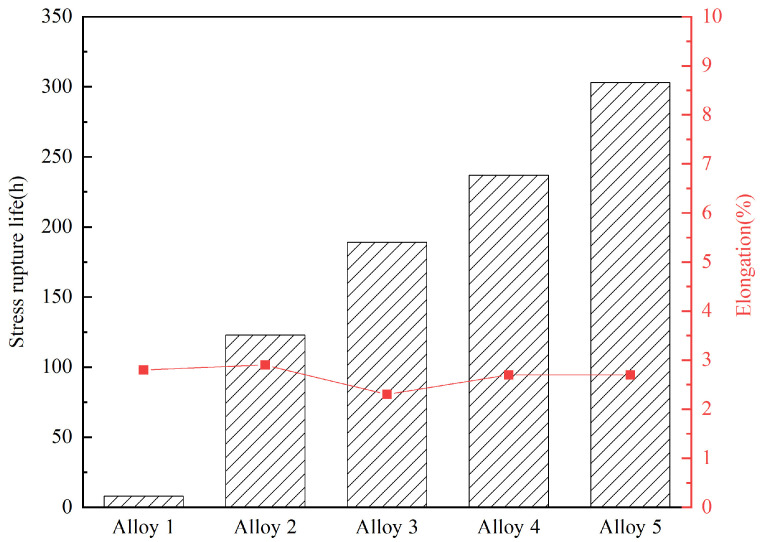
Stress rupture properties of different GH4742 test alloys.

The results of stress rupture properties are given in Table 2 and Figure 5. It was indicated that the addition of B as a single element significantly increased the stress rupture life from 8 h (alloy 1) to 189 h (alloy 3). When a certain amount of RE elements was added to the test alloys, the synergistic effects of B and RE were fully exerted, showing that the stress rupture life had improved to 237 h (alloy 4) and 303 h (alloy 5), respectively. Therefore, a combination of B and RE can significantly improve the stress rupture properties.

**Figure 6:**
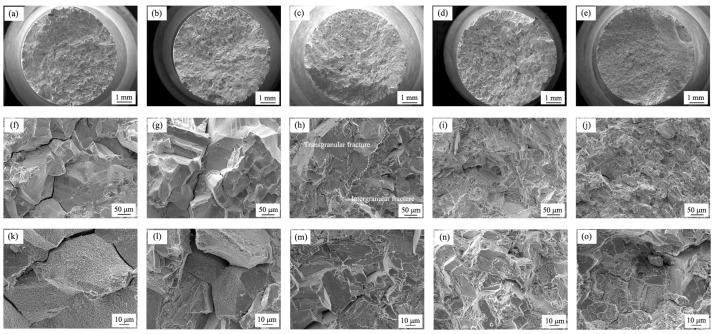
Morphologies of fracture surface of test alloys: (**a**,**f**,**k**) alloy 1; (**b**,**g**,**l**) alloy 2; (**c**,**h**,**m**) alloy 3; (**d**,**i**,**n**) alloy 4; (**e**,**j**,**o**) alloy 5.

Figure 6 shows the fracture surface morphology of the tested stress rupture samples. The failure mode in test alloy 1 and alloy 2 was typically intergranular (Figure 6a,b). The whole surfaces were flat and composed of intergranular cracks. At higher magnification, a large number of secondary cracks were observed on the fracture surfaces, indicating that the strength of the grain boundary was lower than the intergranular strength (Figure 6k,l). In alloy 3, the minor intergranular fracture zone was found around the specimen edge (Figure 6c), while the center part was determined as the transgranular fracture zone with a certain amount of cleavage steps (Figure 6h,m). It was indicated that the mixed fracture mode was dominated in test alloy 3. With the addition of the RE element, the area covered by a transgranular fracture was further expanded in alloy 4 (Figure 6d,e), and, furthermore, the entire surface was fully covered by cleavage steps in alloy 5 (Figure 6j,o). Pictures with high magnification display that there was a small amount of carbon or nitride around the crack source, showing that the cracks begin with precipitates instead of the grain boundary. As a result, the fracture modes converted from intergranular fracture mode to transgranular fracture mode with increasing contents of B and RE due to the effects on improving the grain boundary strength.

## 4. Discussion

### 4.1. Effects of Microstructure on Stress Rupture Properties

Similar to other superalloys, the grain size of the GH4742 alloy under the premise of the same alloying elements is the main issue affecting the tensile properties at room temperature and stress rupture properties at high temperature. Grain refinement is an effective strengthening method in engineering applications. Compared with other strengthening methods, fine grain strengthening is the only way that increases both the strength and plasticity of materials. However, the precondition of fine grain strengthening is that the grain boundary impedes dislocation slip, which exists at a low temperature. For the GH4742 alloy, the grain boundary is essentially a defect when the temperature rises. With the strengthening of atomic activity, the grain boundary also becomes unstable, which will lead to a weakened grain boundary. Therefore, proper grain coarsening is beneficial in improving the stress rupture performance.

Coherent stress strengthening is an important aspect of γ′ phase strengthening, which can influence the dislocation behavior by dispersing in the matrix to achieve alloy strengthening. The γ′ phase, as a strengthening phase for superalloys, has a significant impact on the stress rupture properties. The increase in the content of the γ′ phase and the decrease in size are beneficial for improving the fatigue performance and creep resistance of nickel-based superalloys. The increasing content of the γ′ phase will result in excellent stress rupture properties.

The two influencing factors above play a role in the premise that the alloy composition is unchanged. However, in this study, the influence of alloying elements on the properties is significantly greater than that of these two factors. Therefore, the changes in grain size and γ′ phase are not the main reasons for improving the stress rupture properties.

### 4.2. Effects of B and RE Elements on Stress Rupture Properties

**Figure 7:**
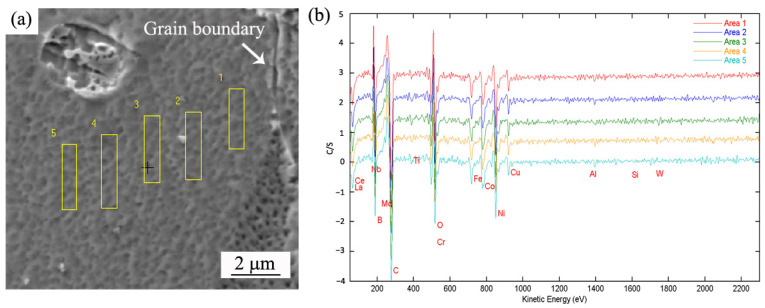
A schematic diagram of selected areas (**a**) and its AES spectrum (**b**) in alloy 5.

Due to the different atomic size and chemical properties between Ni and trace elements, B and RE tend to segregate towards grain boundaries. In order to study element segregation more clearly, five equidistant areas close to the grain boundary were selected for quantitative analysis. A schematic diagram of the selected areas and the AES spectrum are shown in Figure 7. From the results above, the element content was accurately characterized. Table 3 shows the distribution of elements at different positions from grain boundaries in test alloy 5. It can be seen that the content of B in Area 1 near the grain boundary was the highest (6.02%), and the content of B gradually decreased to 4.89% (Area 2), 0.70% (Area 3) and 0.01 (Area 4 and Area 5) as the distance from the grain boundary increased. Although the content of B was not very accurate due to its low addition, it was fully proved that the segregation of B towards the grain boundary was strong. The content of C in Area 1 was also high (13.33%), and the content of C decreased significantly as the distance from the grain boundary increased. The contents of Nb and Ti near the grain boundary were also high, which indicated that carbides precipitated along the grain boundaries. In contrast, the contents of Al, Cr and Co near the grain boundary were much lower than those in the grain, making it clear that these three elements were depleted along the grain boundaries. The contents of Mo and Ti changed minimally. It is noteworthy that no effective contents of RE were detected due to the trace contents.

**Figure 8:**
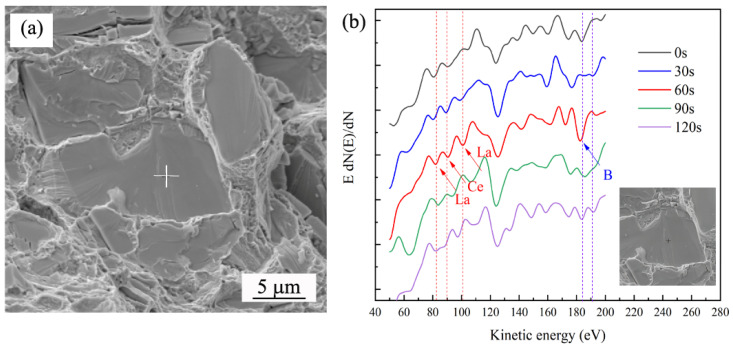
Intergranular fracture morphology (**a**) and AES spectrum of La, Ce and B (**b**) in alloy 5.

Due to the particularity of RE and the fact that the content of RE was not detected in the full spectrum in Figure 7, we designed a specific experiment. A special intergranular fracture surface was prepared, and the variation in the RE and B content sputtered at different times was measured. The AES spectrum in Figure 8 proves the segregation of B and RE elements in alloy 5. Reports have shown that the kinetic energy range of Ce and La is from 52 eV to 115 eV. Typical Auger peaks of La (64 eV, 83 eV and 99 eV) and Ce (68 eV, 87 eV and 104 eV) were chosen for detection. The Auger peak of B was at 185 eV. The change in the inner energy level was caused by the transfer of atomic charge, thus changing the energy of the Auger transition and leading to the displacement of Auger peaks [25,26]. The spectra lines had a minor difference compared to the standard peaks in the Auger Electron Spectroscopy Reference Manual [27].

Due to the existence of high-temperature oxidation, the surface oxygen content was higher when the sputtering time was 0 s and 30 s, and no element segregation was found on the surface. After sputtering for 60 s, some typical peaks of RE and B elements were detected. The tested Auger peak of Ce was at 90 eV, and the tested Auger peaks of La were at 82 eV and 100 eV. Some studies have displayed that RE atoms tend to be enriched at grain boundaries because of their large radius [28,29]. The results above confirmed that Ce and La atoms gathered at the surface of the fracture samples. The same was true for B (the peak was determined at 183 eV), which segregated towards grain boundaries. After sputtering for 90 s, the peak values of peaks disappeared gradually, which indicated that the contents of RE and B elements were decreasing. After sputtering for 120 s, the peaks disappeared totally because the contents of RE and B were too low to detect.

The results above show that the B and RE elements significantly segregated towards the grain boundaries. Moreover, these two kinds of elements usually exist in the form of the atomic state, affecting the distribution of other elements at the grain boundary [15,29]. In the grain boundary regions, where the atomic order is relatively chaotic and the energy is high, B and RE atoms can occupy the vacancies of the grain boundary and improve the interface bonding force, which is considered the main reason for improving the long-time performance.

**Figure 9:**
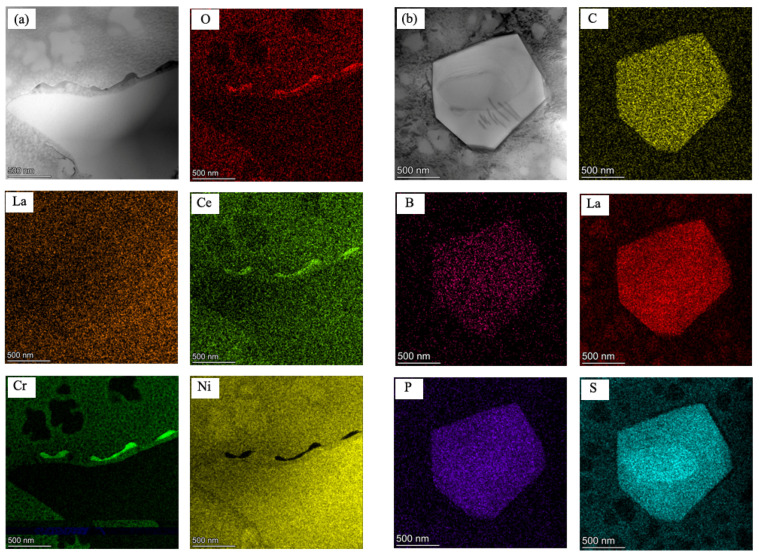
STEM-HAADF images and elemental mapping of grain boundary precipitates in alloy 5 (**a**) RE compound with Ce, O and Cr (**b**) RE complex inclusion with C, B, La, P and S.

Figure 9 manifested two types of precipitates along grain boundaries and their corresponding EDS mappings. The results showed that the nano-scale continuous irregular stirps were enriched with Ce, O and Cr, indicating that the precipitate was an RE compound with a size of less than 500 nm (Figure 9a). Another precipitate was determined as a complex inclusion containing C, B, La, P and S, with an irregular bulk shape (Figure 9b). It was seemingly confirmed that RE elements tend to combine with O and S preferentially, which coincides with the results of a previous study [30,31].

Based on previous research, the nano-scale precipitates had no obvious detrimental effect on the mechanical properties [32,33,34]. When Ce was added to the GH4742 alloy, it seemed to form a compound with a higher melting point, with the original compound showing a lower melting point, thus strengthening the grain boundaries and improving the stress rupture properties.

In addition to stable enrichment at the grain boundaries, La could also form complex inclusions with carbides, which contained P and S elements. P and S were considered to be detrimental impurity elements because of the tendency to segregate towards grain boundaries [35]. Based on the theory of segregation, the impurity elements gathering towards interfaces could weaken grain boundaries and, thus, result in brittle fracture [36,37]. Thereby, the formation of complicated RE precipitates could reduce the segregation of harmful elements towards grain boundaries, playing a purification role and, thus, improving the instantaneous and durable properties of the GH4742 superalloy. However, excess content of La formed Ni5La along the grain boundaries, reducing the stress rupture properties of the alloy based on a previous study [38]. No Ni5La was formed in this study, because the amount of lanthanum was strictly controlled to ensure that it does not exceed the limit.

The atomic radius of B was larger than that of C. The addition of B could reduce the creep rate, improve the sensitivity of permanent notch and increase the high-temperature plasticity of the alloy. The segregation and precipitation of B towards the grain boundaries made it difficult for carbides such as cellular M_23_C_6_ or bulk MC to precipitate and improve the grain boundary state. However, adding too much B may form more B compounds on the grain boundary, which will reduce the workability and plasticity of the alloy [39].

Generally, the segregation of B and RE towards grain boundaries and the synergistic effect with other elements were confirmed based on the investigated data above. As P and S elements were contained in the B and RE precipitates near grain boundaries, the degree of impurity element segregation towards grain boundaries was substantially reduced. This could provide strong evidence that the purification of grain boundaries eliminated the stress concentration, thus leading to an improvement in the stress rupture life. The importance of grain boundary purification was also proved. Therefore, the synergistic effect of B and RE cannot be ignored.

## 5. Conclusions

In this study, the synergistic effects of boron (B) and rare earth (RE) elements on the microstructure and stress rupture properties were studied. The stress rupture properties at 650 °C/873 MPa significantly increased with the addition of B and RE. The grain size and the morphology of the γ′ phase were not the main reasons for improving the stress rupture properties. The enhancement in the stress rupture lifetime was mostly attributed to the segregation of B and RE towards grain boundaries, which increased the binding force of the grain boundary and improved its strength and plasticity. In addition, the enrichment of B and RE inhabited the precipitation of carbides along grain boundaries. Furthermore, two types of RE precipitate containing S and P were found along grain boundaries. The purification of grain boundaries by the B and RE elements was beneficial to the enhancement of stress rupture properties.

The successful development of new GH4742 using the method of microalloying support for the reserve as new materials lays a firm foundation for the development of other superalloys that are hard to deform.

## Figures and Tables

**Table 1:** Chemical compositions of GH4742 alloys (wt.%).

Sample	Cr	Co	Mo	Al	Nb	Ti	Fe	C	B	La	Ce	Ni
Alloy 1	14.09	9.82	5.04	2.73	2.75	2.71	0.031	0.059	0.0005	0.009	0.001	Bal.
Alloy 2	14.04	9.78	5.00	2.71	2.72	2.72	0.065	0.048	0.004	0.009	0.001	Bal.
Alloy 3	13.99	9.90	5.02	2.72	2.66	2.68	0.064	0.048	0.007	0.007	0.001	Bal.
Alloy 4	14.05	9.84	5.06	2.72	2.73	2.68	0.069	0.052	0.007	0.016	0.002	Bal.
Alloy 5	14.03	9.84	5.07	2.76	2.73	2.69	0.078	0.055	0.008	0.034	0.005	Bal.

**Table 2:** Stress rupture properties of GH4742 alloys at 650 °C/873 MPa.

Sample	Stress Rupture Life (h)	Elongation (%)
Alloy 1	8	2.8
Alloy 2	123	2.9
Alloy 3	189	2.3
Alloy 4	237	2.7
Alloy 5	303	2.7

**Table 3:** AES analysis results in test alloy 5 (wt.%).

Element	C	B	Mo	Ti	Nb	Al	Cr	Co	Ni
Area 1	13.33	6.02	3.43	1.24	5.17	0.93	4.61	4.56	Bal.
Area 2	10.49	4.89	3.56	2.33	4.45	1.61	6.57	5.70	Bal.
Area 3	0.57	0.70	3.60	2.34	2.45	1.89	8.83	9.49	Bal.
Area 4	0.12	0.01	4.11	2.24	2.41	2.77	13.76	9.20	Bal.
Area 5	0.093	0.01	4.20	2.51	2.65	2.65	13.58	9.06	Bal.

## Data Availability

Data are contained within this article.
